# Is hepatitis C virus elimination possible among people living with HIV and what will it take to achieve it?

**DOI:** 10.1002/jia2.25062

**Published:** 2018-04-10

**Authors:** Natasha K Martin, Anne Boerekamps, Andrew M Hill, Bart J A Rijnders

**Affiliations:** ^1^ Division of Global Public Health University of California San Diego CA USA; ^2^ School of Social and Community Medicine University of Bristol Bristol United Kingdom; ^3^ Department of Internal Medicine Division of Infectious Diseases Erasmus MC University Medical Center Rotterdam the Netherlands; ^4^ Department of Translational Medicine University of Liverpool Liverpool United Kingdom; ^5^ Department of Medical Microbiology and Infectious Diseases Erasmus MC University Medical Center Rotterdam the Netherlands

**Keywords:** HCV, treatment, elimination, modelling

## Abstract

**Introduction:**

The World Health Organization targets for hepatitis C virus (HCV) elimination include a 90% reduction in new infections by 2030. Our objective is to review the modelling evidence and cost data surrounding feasibility of HCV elimination among people living with HIV (PLWH), and identify likely components for elimination. We also discuss the real‐world experience of HCV direct acting antiviral (DAA) scale‐up and elimination efforts in the Netherlands.

**Methods:**

We review modelling evidence of what intervention scale‐up is required to achieve WHO HCV elimination targets among HIV‐infected (HIV+) people who inject drugs (PWID) and men who have sex with men (MSM), review cost‐effectiveness of HCV therapy among PLWH and discuss economic implications of elimination. We additionally use the real‐world experience of DAA scale‐up in the Netherlands to illustrate the promise and potential challenges of HCV elimination strategies in MSM. Finally, we summarize key components of the HCV elimination response among PWLH.

**Results and discussion:**

Modelling indicates HCV elimination among HIV+ MSM and PWID is potentially achievable but requires combination treatment and either harm reduction or behavioural risk reductions. Preliminary modelling indicates elimination among HIV+ PWID will require elimination efforts among PWID more broadly. Treatment for PLWH and high‐risk populations (PWID and MSM) is cost‐effective in high‐income countries, but costs of DAAs remain a barrier to scale‐up worldwide despite the potential low production price ($50 per 12 week course). In the Netherlands, universal DAA availability led to rapid uptake among HIV+ MSM in 2015/16, and a 50% reduction in acute HCV incidence among HIV+ MSM from 2014 to 2016 was observed. In addition to HCV treatment, elimination among PLWH globally also likely requires regular HCV testing, development of low‐cost accurate HCV diagnostics, reduced costs of DAA therapy, broad treatment access without restrictions, close monitoring for HCV reinfection and retreatment, and harm reduction and/or behavioural interventions.

**Conclusions:**

Achieving WHO HCV Elimination targets is potentially achievable among HIV‐infected populations. Among HIV+ PWID, it likely requires HCV treatment scale‐up combined with harm reduction for both HIV+ and HIV‐ populations. Among HIV+ MSM, elimination likely requires both HCV treatment and behaviour risk reduction among the HIV+ MSM population, the latter of which to date has not been observed. Lower HCV diagnostic and treatment costs will be key to ensuring scale‐up of HCV testing and treatment without restriction, enabling elimination.

## 1 Introduction

Viral hepatitis was the seventh leading cause of death worldwide in 2013, increasing from the tenth leading cause in 2010 1. The vast majority of morbidity and mortality attributable to viral hepatitis is due to hepatitis C virus (HCV) and hepatitis B Virus (HBV). In response to this increasing public health challenge, the World Health Organization recently released targets for HBV and HCV elimination by 2030 (see Table 1) 2. Elimination is traditionally defined as a reduction to zero in the incidence of disease in a specific population or geographical location, with continued prevention efforts required to prevent the re‐establishment of transmission 3. Given this strict definition of elimination would require substantial economic and political resources and could be unattainable in most settings, the goal of “elimination” is often flexibly defined. The recent WHO elimination “as a public health threat” targets are comprised of a 90% relative reduction in new infections and a 65% relative reduction in hepatitis‐related mortality by 2030.

**Table 1:** WHO hepatitis elimination goals: impact and service coverage targets 2

	WHO TARGET BY 2030
Impact Targets	
Incidence: New cases of chronic HBV and HCV	90% relative reduction
Mortality: HBV and HCV deaths	65% relative reduction
Service Coverage Targets	
HBV childhood vaccination coverage	90%
HBV birth dose vaccination coverage or other PMTCT initiative	90%
Screening of blood donations	100%
Safe injections: % of injections administered with safety engineered devices in and out of health facilities	90%
Harm reduction: number of sterile needles and syringes provided per person who inject drugs per year	300
HBV and HCV diagnosis	90%
HBV and HCV treatment	80% of persons with chronic infection treated

PMTCT, prevention of mother to child transmission.

Hence, to achieve the WHO incidence elimination targets, efforts must focus on both prevention of *disease* and prevention of *transmission*. The advent of highly effective HCV direct acting antiviral (DAA) therapy, with sustained viral response (SVR) exceeding 90% in both HCV monoinfected and HIV/HCV coinfected populations 4, 5, 6, 7, 8 has renewed optimism that substantial reductions or elimination of end stage liver disease and HCV‐related mortality is a possibility. The widespread use of HIV antiretroviral treatment for prevention has also led to speculation that HCV treatment could also be used for prevention. In addition, among people who inject drugs (PWID), a key risk group for HCV, harm reduction interventions such as opiate substitution therapy (OST) and needle and syringe programs (NSP) have been the traditional backbone of HCV prevention. A recent Cochrane systematic review 9 found that OST reduces risk of HCV acquisition by 50% (risk ratio 0.50 95% CI 0.40 to 0.63) and combined with high coverage of NSP results in a 71% reduction in the risk of HCV acquisition (Risk Ratio = 0.29 95% CI = 0.13 to 0.65).

The WHO strategy does not include discussion relating to elimination of viral hepatitis among HIV‐infected populations specifically. Yet, because of the shared transmission routes many people living with HIV (PLWH) are coinfected with viral hepatitis. Globally, approximately 6.2% (3.4% to 11.9%) of PLWH are coinfected with HCV, equating to approximately 2.28 million (IQR 1.27 to 4.42) HIV/HCV coinfected individuals 10. Indeed, PLWH are six times more likely to be infected with HCV compared to those not infected with HIV. The burden of HIV‐HCV coinfection is particularly high among high‐risk groups such as men who have sex with men (MSM) and PWID. Among HIV‐infected individuals worldwide, it has been estimated that 2.4% (IQR 0.8 to 5.8) are coinfected with HCV within general population samples, yet this rises to 6.4% (3.2 to 10.0) in MSM, and 82.4% (55.2 to 88.5) in PWID 10.

As such, to achieve the WHO HCV elimination incidence reduction target among PWLH it is crucial to tackle transmission among HIV‐infected MSM and PWID. However, although the prevention interventions required to eliminate HCV globally will be equally applicable to coinfected populations, the intervention level required and targeting may be different based on specific epidemic characteristics.

The objective of this paper is to review the modelling and cost evidence surrounding feasibility of HCV elimination among HIV‐infected (HIV+) key populations such as MSM and PWID and identify the likely components required for HCV elimination among PLWH. We use the real world experience of HCV DAA scale‐up in the Netherlands through the Dutch Acute HCV in HIV Studies to illustrate the promise and potential challenges of HCV elimination strategies in a key population (MSM).

## 2 Methods

This analysis is comprised of four parts: (i) A review of the theoretical mathematical modelling literature examining what prevention and treatment scale‐up is required for HCV elimination among HIV‐infected PWID and HIV‐infected MSM populations. (ii) A review of the cost‐effectiveness of HCV treatment for HIV‐infected populations and discussion of cost considerations for elimination. (iii) A discussion of the real‐world experience of HCV DAA scale‐up among HIV+ MSM in the Netherlands (iv) A summary of probable and possible components of the HCV elimination response among PWLH.

## 3 Results and discussion

### 3.1 Modelling the scale‐up needed for HCV elimination among HIV‐infected populations

#### 3.1.1 HIV‐infected PWID

Numerous burden of disease models have shown that existing or modestly increased levels of treatment targeted at individuals with more advanced liver disease can achieve the WHO HCV mortality target (65% reduction by 2030) in a variety of global settings. For example, a regional European Union model showed that HCV treatment only need to increase from 150,000 patients in 2015 to 187,000 patients in 2025 to achieve the mortality elimination target 11. A separate multi‐country analysis including some resource limited settings found that achieving the WHO HCV mortality target in Hungary, Indonesia, Lebanon, Pakistan and Romania is unlikely to be achieved with existing screening/treatment programs, but could be achieved with scaled‐up screening and treatment 12. These models of disease progression are particularly valuable in identifying the level and targeting of treatment required to reduce HCV mortality, but because they do not mechanistically incorporate disease transmission are unable to shed light on what is required to achieve the WHO incidence elimination target.

A wide body of literature since 2011 has utilized epidemic modelling to explore what level of prevention scale‐up could result in control and elimination among PWID, and whether HCV treatment could be used for prevention. Several initial modelling studies in the UK and general PWID populations of varying prevalences have indicated that harm reduction alone is unlikely to achieve HCV elimination among PWID populations 13, 14. Subsequent studies have explored the potential of HCV treatment as prevention among PWID populations in a range of settings including North America, Europe, Asia and Australia 13, 15, 16, 17, 18, 19, 20. Broadly speaking, these studies have generally found that scale up of HCV treatment to rates to below 100 per 1000 PWID annually, particularly in combination with harm reduction 13, 16, 21, can reduce HCV incidence by 90% by 2030 across a wide range of settings. Results have been consistent between high and low income settings examined, such as Vietnam 20.

In addition, modelling studies have pointed to several key additional elements which are required for achieving elimination among PWID: One Australian study highlighted the need for enhanced HCV screening among PWID in order to sustain HCV treatment rates required for elimination, a situation which is likely applicable to many global settings 22. In addition, a modelling study in the rural U.S. emphasized the importance of retreatment of reinfection in achieving elimination targets 21. As such, elimination strategies likely require regular testing 22, HCV treatment 13, 15, 16, 17, 18, 19, 20, harm reduction 13, 16, 21 and retreatment of reinfections 21.

Despite several studies modelling HIV and HCV coinfection transmission among PWID 23, 24, to our knowledge as of 2017 no published study has explored what is required for HCV elimination among HIV‐infected PWID in particular. Preliminary modelling presented at a recent international conference indicated that HCV treatment targeted at HIV‐infected PWID in Andalusia, Spain would not achieve elimination among this population due to continued risk of HCV transmission from HIV‐negative PWID populations 25. Therefore, more generally, because the burden of HCV is high among HIV‐uninfected PWID populations, and as HIV‐infected PWID populations are likely to mix with HIV‐uninfected PWID populations, elimination among HIV‐infected PWID is probably only achievable if combination HCV prevention efforts are targeted both HIV+ and HIV‐ PWID populations.

#### 3.1.2 HIV‐infected MSM

An HCV epidemic among HIV‐infected MSM has been observed in the United States, Western Europe, Australia, Taiwan, Hong Kong and Japan, with HCV incidence and prevalence among HIV‐infected MSM substantially higher than the HIV‐uninfected MSM population 26, 27. A growing number of modelling studies since 2015 have explored what level of intervention (treatment and/or risk reduction) is required to eliminate HCV among HIV‐infected MSM populations 28, 29, 30, 31. To date, these studies have focused exclusively on Western European settings (UK, Switzerland, Berlin and the Netherlands), and no studies have explored resource limited settings, in part because of the lack of epidemiological data on HCV epidemics among HIV+ MSM in these settings. However, the specific epidemic characteristics between the modelled settings has varied. One unifying characteristic is the relatively low rates of primary incidence among HIV+ MSM (1 to 2 per 100 person‐years 26, 32) compared to PWID populations but high rates of reinfection (2 to 10 fold that of primary incidence 33, 34, 35). Together these could pose a challenge for elimination efforts, where a relative reduction in 90% would translate to very low targets (0.1 to 0.2/100 person‐years) which could be particularly hampered by high rates of reinfection. Nevertheless, the absolute numbers of HCV‐HIV coinfected MSM are small, most diagnosed HIV+ MSM are linked to care in high‐income settings where HCV epidemics among HIV+ MSM have been documented 36, 37, 38 so HCV elimination may be particularly feasible in this group.

As with PWID, modelling indicates the level of intervention required among HIV+ MSM to achieve the WHO elimination targets varies by epidemiological setting, particularly given substantial variation in incidence trends. For example, among HIV+ MSM incidence of HCV over the past decade has remained relatively stable in the UK and the Netherlands. In the UK, a modelling study indicated that scaled‐up rates of DAA therapy (from 46% to 80% treated within a year of diagnosis and from 7%/year to 20%/year thereafter) could reduce incidence among HIV+ MSM over 60% by 2030, but could not meet elimination targets 28. Elimination targets could be reached when all those diagnosed receive treatment within 1 year of diagnosis, or if treatment scale‐up is combined with a behavioural risk reduction 25.

In contrast, HCV incidence among HIV+ MSM has steadily increased over the past decade in Switzerland and Germany. In Switzerland, this has occurred alongside an increase in self‐reported risk behaviour (unprotected anal intercourse). A recent modelling study in Switzerland projected that if these trends continue, elimination (or even reductions in HCV incidence) could not be achieved through HCV treatment alone, and requires additional reduction in high‐risk behaviour (perhaps through behavioural interventions) 29. Preliminary modelling in Berlin supports the Swiss findings. Germany is a unique setting in that universal access for DAAs has been available since 2014, and as such relatively high‐treatment rates (>80% HIV+ MSM treated after their acute diagnosis) have been achieved. However, the continued increase in HCV incidence among HIV+ MSM in Berlin and Germany overall (from 0.33/100 person‐years in 1996 to 1999 to 2.28/100 person‐years in 2008 to 2012 in Germany 39) and high levels of reinfection (7 to 8 per 100 person‐years 33) mean that elimination by 2030 likely requires both further scale‐up of HCV treatment and reductions in high‐risk behaviour 31.

Finally, preliminary modelling in the Netherlands indicated immediate treatment of all diagnosed HIV+ MSM with DAAs could only result in moderate reductions in HCV incidence among HIV+ MSM (approximately 30% within 15 years), but not reach WHO elimination targets 30. However, the real‐world observation of a halving of HCV incidence among HIV+ MSM from 2014 to 2016 with expansion of DAA therapy as described below has raised excitement about the potential for elimination via treatment as prevention among HIV+ MSM in the Netherlands, described later in this paper.

### 3.2 Cost and cost‐effectiveness implications of HCV testing and treatment scale‐up for elimination among HIV‐infected populations

There is a wide body of evidence that HCV treatment is cost‐effective for HIV‐infected populations, including HIV+ MSM with a risk of reinfection, 40, 41, 42, 43, 44 in high‐income settings such as the United States and the UK. In addition, as mentioned previously, achieving HCV elimination among key risk groups such as HIV+ PWID, may require targeting the broader PWID population. Numerous economic evaluations have shown that HCV treatment is cost‐effective for PWID populations in high‐income settings 45, 46, 47, 48, 49, 50, 51, 52, 53 despite the potential risk of reinfection and higher mortality rates among PWID populations. Indeed, economic evaluations indicate treating PWID with an ongoing risk of transmission may accrue substantial economic benefits through prevention of transmission. In addition, an economic analysis in Australia found that HCV treatment scale‐up to achieve the WHO targets among PWID was cost‐effective 15. Unfortunately, no cost‐effectiveness studies for HIV‐infected populations or PWID have been performed in resource limited settings. However, economic analyses have shown that DAA therapy for the general population is cost‐effective India 54 and Egypt 55, 56 where generic or low cost DAAs are available.

Despite the evidence HCV treatment is cost‐effective, the high costs of HCV treatment 57 (>$75,000 per 12 week treatment course for sofosbuvir+daclatasvir in the US and UK) remain a major barrier to HCV treatment scale‐up. Prices vary widely depending on country and income status 57, and prices of innovator and generic medicines have fallen, but nevertheless remain prohibitively high for widespread scale‐up in developed and developing countries alike.

Unfortunately, the high costs of HCV treatment have so far resulted in prioritization of HCV therapy (or restrictions on insurance reimbursement) even in developed countries 58, 59. In these settings, patients with more advanced liver disease and those coinfected with HIV have traditionally been prioritized for early treatment. Although this type of strategy will be effective in preventing HCV‐related morbidity and mortality among PWLH, it undermines elimination efforts as PLWH will remain at risk of being infected or reinfected with HCV from individuals who remain untreated and are at risk of transmitting. For example, PWID with a risk of transmission tend to be younger with less advanced liver disease, and therefore prioritization strategies targeting individuals with advanced liver disease may fail to prevent the substantial amount of transmission from this group 60. This is despite economic assessments indicating that treatment scale‐up among PWID at or below the level required for elimination is cost‐effective in settings like Australia, the UK and Netherlands 15, 60, 61. In addition, models indicate early treatment for PWID is cost‐effective compared to delay until cirrhosis, and may be more cost‐effective than early treatment for those with no ongoing risk in settings with low‐moderate (20/40%) HCV prevalence among PWID due to substantial prevention benefits of early treatment of PWID 60. Unfortunately, no economic evaluations have assessed whether scaled‐up treatment to achieve HCV elimination among PWID is cost‐effective in resource‐limited settings.

However, although HCV therapy is likely cost‐effective, the high costs per treatment results in a substantial budgetary impact in countries with a large HCV infected populations. This has resulted in HCV treatment restrictions even in resource rich countries in the U.S. and Europe 58, 59. Indeed, HCV treatment coverage is still low globally 62. A recent analysis estimated that the percentage of people with HCV who were treated with DAAs in 2016 ranged from 8.1% in North America and North Africa/Middle East to 0.1% in sub‐Saharan Africa 63. Among 91 countries analyzed, 47 countries had more new HCV infections than individuals who achieved cure through HCV treatment in 2016 63, indicating that these countries are failing to turn off the tap of new infections with treatment. Nevertheless, some countries are achieving very high‐treatment rates among specific sub‐populations, such as among HIV+ MSM in the Netherlands, discussed in the next section.

Promisingly, HCV DAA therapies could be produced as generics at a fraction of the current costs 64, particularly from within a country such as India due to its sizeable generic industry and low production costs. A recent analysis estimated the costs of generic HCV DAA production based on the costs of their active pharmaceutical ingredients. This analysis found that the combination treatment sofosbuvir and daclatasvir has an estimated generic cost of $50 to 72 per 12‐week course with a 10% to 50% profit margin (Hill A, unpublished results).

In addition, even the costs of HCV diagnosis and monitoring remain prohibitive in many developing countries 65. For example, in India, generic HCV treatments are available for at or below $300 per treatment course, yet HCV antibody and RNA testing costs an estimated $17 and $108, respectively 66. In addition, with current treatment monitoring as suggested by the Indian national guidelines 67 (every 4 weeks with RNA tests at week 0, 12, and SVR12) the cost of treatment delivery could easily far exceed that of HCV treatment.

Finally, in many settings additional financing will be required to build the capacity of health services for diagnosis and treatment of HCV. However, the economic implications of this health systems strengthening (in terms of increased personnel, training, infrastructure, etc.) has not been estimated. It is possible that integration of HCV testing and treatment within HIV services will prove to be an effective and cost‐effective approach 68, 69.

### 3.3 HCV elimination among HIV‐infected MSM in the real world: Dutch experience

In the Netherlands, surveillance data indicate that among PLWH, the vast majority of acute HCV infection diagnoses occur among MSM 70. In this section, we detail the Dutch experience of HCV direct‐acting antiviral (DAA) scale‐up and impact on acute HCV incidence and HCV prevalence among HIV+ MSM.

HIV and HCV care is well‐organized in the Netherlands. All patients diagnosed with HIV are cared for by a team of infectious diseases physicians and specially trained HIV nurses in 26 treatment centres spread across the country. Screening for chronic HCV is universal at entry into HIV care and more than 99% of the HIV‐infected patients in care in the Netherlands have been tested for HCV at least once 71. Screening for incident HCV infections in MSM is performed by testing ALT (followed by HCV testing when a new ALT elevation is observed) twice a year. HCV/HIV coinfected MSM visit the HIV outpatient clinic at least twice a year. HCV infections can be treated by the infectious diseases physician so no referral to a hepatologist is needed. Facilitated by specially trained on‐site data collectors, detailed clinical and laboratory data from consenting patients (98%) are registered in a central database, comprising the AIDS Therapy Evaluation in the Netherlands (ATHENA) cohort. In addition, two prospective acute HCV treatment studies among HIV+ individuals have occurred (Dutch Acute HCV in HIV Studies, DAHHS1 (from 2013 to 2014) and DAHHS2 (2016‐ongoing)) 72, 73 in 17 centres providing care for 75% of the Dutch HIV+ MSM population.

In the Netherlands, DAAs were available from September 2014 for HCV‐infected patients with significant liver fibrosis or cirrhosis, and October 2015 regardless of fibrosis stage. This led to a very rapid uptake of HCV therapy among HIV+ MSM in the ATHENA cohort, with 79% attaining SVR just 14 months after restrictions were lifted 74 and a substantial decrease in the pool of HCV RNA positive HIV‐infected MSM in care in the Netherlands. Indeed, while in 2015 4.1% (450 of 11070) of the HIV+ MSM in care were HCV RNA+, this decreased to 1.5% (176 of 11749) by the end of 2016. As of May 2017, less than 150 HCV infected HIV+ MSM remain to be treated. With only 1.5% of the HIV+ MSM population currently remaining HCV infected, HCV elimination may become a reality. However, the residual group of infected patients is likely a more difficult to reach sub‐group, and the risk of reinfection among HIV‐positive MSM is high 33, 34, 75, 76.

The data described above demonstrate that a very rapid decline in the *prevalence* of HCV can be achieved in a well‐organized healthcare system of a resource rich country. However, to achieve HCV elimination according to the WHO targets, the *incidence* of new HCV infections needs to decrease by 90% by 2030 2. We obtained data from the DAHHS studies to compare the incidence of acute HCV infections in the first year after universal DAA availability (2016) with the last year before DAA became available (2014). From 2014 to 2016, a 51% decrease in acute HCV infections was observed 77. Furthermore, this decrease contrasted with a significant increase in the percentage of positive syphilis (+2.2%) and gonorrhoea (+2.85%) tests in HIV‐positive MSM observed at STD clinics across the country 78, 79, 80, indicating that the reduction in HCV was unlikely to be due to behavioural risk reduction.

While the substantial reduction in HCV incidence among HIV+ MSM observed after widespread scale‐up of HCV treatment in the Netherlands is reason for optimism, an observational study cannot prove a causal relationship. Further modelling work will be required to disentangle the estimated impact of HCV treatment as prevention initiatives among HIV+ MSM in the Netherlands, and coverage required for elimination. We consider it unlikely that HCV treatment as prevention alone will result in a 90% reduction in the incidence of HCV (the WHO target) among HIV+ MSM in the Netherlands, and discuss additional steps likely required in the following section. Indeed, no further decline in the HCV incidence has been observed in 2017 so far 81. In addition, we note that the Dutch experience is a specific example within a resource‐rich country with well‐coordinated HIV and HCV care. Whether the experience in the Netherlands will translate to other resource‐limited settings requires further study.

### 3.4 Probable and possible components required to achieve HCV elimination among PLWH

In summary, despite the promise of HCV elimination using HCV treatment as prevention from both theoretical modelling studies and real‐world observations in the Netherlands, numerous *probable* and *possible* barriers exist which could hamper HCV elimination efforts among PLWH (Table 2). As such, the following components are likely an important part of the HCV elimination response among PWLH (based on *probable* barriers):

1. Regular HCV testing of high‐risk populations, both HIV+ and HIV‐: Among HIV+ PWID, modelling indicates that elimination likely requires elimination efforts among the broader PWID population 25, yet worldwide an estimated 80% of HCV‐infected individuals remain undiagnosed 62, a situation which may be worse among PWID. For example, in India only an estimated 5% of HCV‐infected PWID are diagnosed 82. Consequently, the treatment scale‐up required for elimination among PWID likely requires enhanced testing among both HIV+ and HIV‐ PWID 22. Among HIV+ MSM, modelling indicates that regular testing of HIV+ MSM is also likely required for elimination. Testing of other MSM populations is described below under possible barriers.
2. Development of low cost, simple, reliable and accurate HCV diagnostics. Even in low‐income countries with low cost DAAs, the price of HCV diagnostics remains a barrier 65. In addition, the currently available diagnostic products are complex, many which require a cold chain and/or show poor accuracy among HIV‐infected individuals 83.
3. Reduced costs for DAA treatment. Despite the availability of generic and low‐cost DAAs in some resource limited settings, costs of DAA therapy in the vast majority of countries remains a barrier to widespread scale‐up of HCV treatment 57, 84, 85. Greater market transparency and price negotiations are required 84.
4. Broad access to HCV treatment without restrictions: Modelling studies indicate that restricting treatment for those with more advanced fibrosis and/or by drug use status, as is occurring in many settings 58, 59, will likely have limited impact on preventing transmission among PWID populations 86, the vast majority who are younger with less advanced disease. In settings where HCV epidemics are predominantly PWID‐driven, broad access to HCV treatment regardless of disease stage is therefore required for HCV elimination. Even in settings with substantial general population transmission, it is likely that restricting treatment for more advanced disease stages will mean that substantially more treatments are required to achieve elimination 87.
5. Close monitoring for reinfection and retreatment of reinfections: Treating those at risk of transmission, the target group for HCV treatment as prevention efforts, will result in reinfections. Among PWID, lower rates of reinfection compared to primary infection were reported in the IFN‐era 88, and reinfection rates among PWID on OST in DAA trials have been low (<3 per 100 person‐years) 89. However, modelling in a rural expanding epidemic setting in the United States indicates achieving the WHO elimination incidence target among PWID requires retreatment of reinfections 21. Among HIV+ MSM, European studies indicate high incidence of HCV reinfection in HIV+ MSM (2 to 10 fold that of primary infection rates) in both the IFN‐era 33, 34, 76 and DAA era 75. As modelling studies have shown that more frequent testing for HCV and earlier initiation of HCV treatment could reduce the HCV epidemic among HIV‐positive MSM 31, 90 and given that those previously infected with HCV are a particularly high risk sub‐population for transmission, reducing the time from reinfection to retreatment is important. This could be achieved by increasing the frequency of HCV reinfection screening and may require out of the box diagnostic strategies like a home‐based diagnostic approach in which the patient collects dried blot spots that are sent to the lab for HCV RNA or antigen testing.
6. Harm reduction and other behavioural interventions to prevent infection/reinfection: Despite the importance of harm reduction such as OST and needle and syringe programs for preventing HCV infection among PWID 9, access to these interventions in many settings is poor, particularly in many resource limited settings 91. Scale‐up of harm reduction, as recommended by the WHO 2, is crucial. In addition, among HIV+ MSM, effective behavioural interventions to prevent HCV infection are urgently required, as modelling from several settings indicates elimination of HCV in this population without effective behavioural intervention will be unlikely 28, 29, 31. Unfortunately, there is a lack of robust evidence surrounding the efficacy of behavioural change interventions targeting HCV risk among HIV+ MSM. It is possible that some interventions developed to prevent HIV transmission among MSM may also be effective against HCV, particularly those targeting unprotected anal intercourse 29, 92. There is an emerging body of literature examining the development of educational and counselling interventions targeted at MSM who engage in ChemSex 93, 94, 95, which may reduce the risk of acquiring HCV among this population. Further research is needed examining the development, acceptability, and efficacy of culturally sensitive behavioural change interventions for preventing HCV infection.

**Table 2:** Barriers to HCV elimination among PLWH

Probable barriers to HCV elimination among PLWH:
• Low levels of diagnosis in many settings and risk populations 62
• Lack of availability of low cost, simple, reliable and accurate HCV diagnostics for LMIC settings 65, 83
• High costs of DAA treatment 57
• Restrictions on DAA accessibility by fibrosis stage and drug use status 58, 59
• Low levels of harm reduction availability for PWID in many settings 91 • High reinfection incidence among HIV+ MSM 33, 34, 75, 76 • Lack of evidence‐based interventions to reduce HCV infection among HIV+ MSM
Possible additional barriers:
• Transmission of HCV from HIV uninfected MSM, such as those on PrEP
• Spread of HCV clones with acquired DAA resistance 100 • Lack of licensing for HCV treatment in the acute stage • Cross border transmission of HCV, particularly among HIV+ MSM 97, 98 and between countries with different levels of DAA availability

In addition, the following items would address *possible* barriers to elimination and therefore may be required for elimination among PWLH. We note that further evidence is required to support whether these items are necessary components of an elimination response:

1. Testing among HIV‐negative MSM receiving HIV pre‐exposure prophylaxis (PrEP): There is growing evidence of HCV infection among HIV‐negative MSM receiving PrEP, a group which could contribute to HCV transmission to HIV+ MSM. In Amsterdam, the prevalence of chronic HCV among HIV‐negative MSM in a PrEP implementation programme was 4% (15/375), comparable to the prevalence of 4.2% in HIV+ MSM 96. In Antwerp, Belgium, HCV prevalence among HIV‐negative MSM at the start of a PrEP project was 2%, and several new HCV infections were diagnosed during follow‐up in these PrEP users (Rijnders B, personal communication). The extent to which HIV‐negative MSM (particularly on PrEP) contribute or will contribute to the HCV epidemic among HIV+ MSM in unclear, but increased epidemiological surveillance will shed light on the burden of disease in this group and help understand their potential importance to the HIV+ MSM epidemic.
2. Coordinated multi‐country prevention and treatment effort: Among HIV+ PWID, the contribution of cross‐border transmission and import/export of infections to the epidemic is unclear. Among HIV+ MSM, the highly connected nature of the HCV epidemic among HIV+ MSM in Western Europe and frequent travel of MSM between European cities has been documented 97, 98. Therefore, at least in Europe, local elimination of HCV among HIV+ MSM may require coordinated multi‐country efforts.
3. Licensing for treatment in the acute stage: Currently none of the approved DAA regimens are licensed for the treatment of acute HCV. Depending on the regional legislations, this may result in a compulsory “wait for documented chronicity” policy and thus increase the duration of time for onwards HCV transmissions to occur. HIV modelling studies have highlighted the importance of the acute HIV stage on the HIV epidemic 99. However, the importance of acute HCV infection in relation to the HCV epidemic among PLWH is unclear and further research is needed to determine to what degree and in what populations early treatment is required for achieving elimination targets.
4. Monitor if transmission of HCV clones with acquired DAA resistance occurs: It was recently shown that the HCV Q80K polymorphism, associated with DAA resistance, is frequently detected and transmitted among HIV+ MSM in the Netherlands 100. As relapse with documented DAA resistance is rare, the chance of a patient developing ánd transmitting acquired DAA resistance is theoretically small and as far as we know has only been described in the context of treatment with a first generation protease inhibitor 101. As such, further research is needed to ascertain the importance of resistance in the DAA era.

## 4 Conclusions

Both theoretical modelling studies and emerging real world evidence from the Netherlands indicate that HCV elimination among HIV‐infected key populations such as MSM and PWID is potentially achievable. Due to a number of factors, elimination of HCV among HIV+ MSM may be complex; and require both high coverage HCV treatment and behavioural interventions. Elimination among HIV+ PWID will likely require elimination efforts among PWID more broadly. In addition to HCV treatment as prevention initiatives, elimination among PLWH also likely requires regular HCV testing, development of low‐cost accurate HCV diagnostics, reduced costs of DAA therapy, broad treatment access without restrictions, close monitoring for HCV reinfection and retreatment, and harm reduction and/or behavioural interventions.

Finally, we note that the vast majority of existing research is limited to high‐income settings, and more research is required relating to HCV elimination among PLWH in resource limited settings. For example, more modelling work is needed to assess what scale‐up is required for HCV elimination among PLWH populations in resource‐ limited settings where transmission routes may vary and the HCV epidemics among HIV‐infected populations may differ. Many developing countries have high burdens of injecting drug use as well as high HCV among HIV‐infected PWID 10, 102. Yet, their risks may be different from high‐income settings. For example, an increasing number of analyses are focusing on HCV elimination among the general population in low or middle‐income country settings such as Georgia and Pakistan 87, 103, which have shown that even in settings with high numbers of PWID, transmission may be highly disseminated, with PWID experiencing risk both from injecting drug use and the broader community through iatrogenic transmission. Hence, the requirements for elimination in these settings where PWID experience multiple risks may be different than in other settings. In addition, data are lacking on HCV among MSM in resource‐limited settings, and as such the magnitude of the problem and requirements for elimination are unclear. In addition to differences in epidemic characteristics, clearly economic considerations across resource‐limited countries will vary substantially, and as such the requirements for achieving HCV elimination among PWLH in these resource limited settings requires further study.
